# Complex Structure of Engineered Modular Domains Defining Molecular Interaction between ICAM-1 and Integrin LFA-1

**DOI:** 10.1371/journal.pone.0044124

**Published:** 2012-08-30

**Authors:** Sungkwon Kang, Chae Un Kim, Xiaoling Gu, Roisin M. Owens, Sarah J. van Rijn, Vanissra Boonyaleepun, Yuxin Mao, Timothy A. Springer, Moonsoo M. Jin

**Affiliations:** 1 Department of Biomedical Engineering, Cornell University, Ithaca, New York, United States of America; 2 Cornell High Energy Synchrotron Source, Cornell University, Ithaca, New York, United States of America; 3 Department of Molecular Biology and Genetics, Cornell University, Ithaca, New York, United States of America; 4 Immune Disease Institute and Department of Pathology, Harvard Medical School, Boston, Massachusetts, United States of America; University of Oulu, Finland

## Abstract

Intermolecular contacts between integrin LFA-1 (α_L_β_2_) and ICAM-1 derive solely from the integrin α_L_ I domain and the first domain (D1) of ICAM-1. This study presents a crystal structure of the engineered complex of the α_L_ I domain and ICAM-1 D1. Previously, we engineered the I domain for high affinity by point mutations that were identified by a directed evolution approach. In order to examine α_L_ I domain allostery between the C-terminal α7-helix (allosteric site) and the metal-ion dependent adhesion site (active site), we have chosen a high affinity variant without mutations directly influencing either the position of the α7-helix or the active sites. In our crystal, the α_L_ I domain was found to have a high affinity conformation to D1 with its α7-helix displaced downward away from the binding interface, recapitulating a current understanding of the allostery in the I domain and its linkage to neighboring domains of integrins in signaling. To enable soluble D1 of ICAM-1 to fold on its own, we also engineered D1 to be functional by mutations, which were found to be those that would convert hydrogen bond networks in the solvent-excluded core into vdW contacts. The backbone structure of the β-sandwich fold and the epitope for I domain binding of the engineered D1 were essentially identical to those of wild-type D1. Most deviations in engineered D1 were found in the loops at the N-terminal region that interacts with human rhinovirus (HRV). Structural deviation found in engineered D1 was overall in agreement with the function of engineered D1 observed previously, i.e., full capacity binding to α_L_ I domain but reduced interaction with HRV.

## Introduction

Integrins are noncovalently associated αβ heterodimeric cell surface receptors that mediate cell-cell and cell-extracellular matrix adhesions, signaling bidirectionally across the plasma membrane. Integrins play important roles in development, immune cell trafficking and responses, and homeostasis [1], [2], [3]. One of the major leukocyte integrins is the lymphocyte function-associated antigen (LFA)-1, which provides the interactions necessary for immunological synapse formation and adhesion to endothelial cells [4]. Ligands of LFA-1 include intercellular adhesion molecules (ICAMs; ICAM-1, -2, -3, -4, and -5) [5] and junctional adhesion molecule (JAM)-1 [6], both of which are the members of the immunoglobulin superfamily (IgSF) receptors. As one of the most biologically important ligands for LFA-1, ICAM-1 is expressed at a low constitutive level in diverse types of cells and tissues, while its expression is greatly upregulated in response to inflammation [7] and in some tumors and their stroma [8], [9], [10], [11], [12], [13], [14]. The interaction of LFA-1 and ICAM-1 is contained within the single domains called the α I domain in LFA-1 and the first N-terminal domain (D1) of ICAM-1. ICAM-1 is also subverted as a receptor for human rhinovirus (HRV): the epitopes for both HRV and LFA-1 are within D1, yet they are distinct [15].

Previous structural studies have indicated that the I domains of both α and β chains exhibit low to high affinities to their ligands [16], [17]. Distinct conformational changes have been observed between the top of the I domains known as the metal ion-dependent adhesion site (MIDAS) and the C-terminal α-helix (designated as α7-helix), a molecular coupling characterized as ‘allostery’. The displacement of the α7-helix (allosteric site) ‘downward’ (with respect to the top defined as the binding interface of the I domain with ligands) has been hypothesized to cause a change in the coordination to the metal ion of the residues in the MIDAS (active site), leading to a higher affinity conformation [18]. For integrins containing I domains in the α subunits, the downward ‘pull’ of the α7-helix is coupled to global conformational rearrangements of integrins, and more specifically, to the opening of the integrin headpiece and the separation of α and β subunits at the plasma membrane [19]. Structural change in the integrin is linked to its bidirectional cell signaling across the plasma membrane, termed “outside-in” and “inside-out” signaling [1]. The LFA-1 α I domain is functionally expressed in isolation, but is dominantly in a low affinity conformation to physiologic ligands. A structural linkage between the MIDAS and the α7-helix, *i.e.* allostery in α I domains, has been demonstrated by rationally designed mutational studies [17], [20] and by a molecular simulation study [21]. However, no previous crystal structures of α I domains in complex with physiological ligands were obtained with the native sequence in the α7-helix and the residues in contact with the α7-helix. Compared to the rationally designed activating mutations in the LFA-1 α I domain, we previously reported an application of directed evolution to select active I domains from a library through a selective pressure for binding to ICAM-1 [22]. Several point mutations away from the allosteric site within and in the vicinity of the α7-helix were identified, which induced higher affinity to ICAM-1.

The ectodomain of ICAM-1 contains five Ig-like domains with the first domain D1 solely responsible for interactions with LFA-1 and HRV. The binding sites in D1 for LFA-1 and HRV, however, are distinct. The loops at the N-terminal face of D1 interact with HRV by docking into a region known as the canyon [15], [23], whereas the residues within the β-strands make contact with the I domain [17]. Despite the modular nature of many IgSF domains, D1 does not fold on its own unless it is expressed with the second domain D2 [15], [24]. In an attempt to achieve a physiologic fold, we have previously engineered D1 by directed evolution [25]. A set of extensive and concurrent mutations in D1 were necessary to express D1 on its own that is competent for binding to the LFA-1 I domain and conformation-specific antibodies. We have previously noted that D1 contains a hydrogen bond network in the core of the domain and that the mutations selected for the native conformation were mainly those converting hydrogen bond interactions to hydrophobic, van der Waals (vdW) contacts. Engineered D1 retained an interaction with the I domain, comparable to the wild-type ICAM-1. However, full-length ICAM-1 containing the mutations found in D1 exhibited lower binding to HRV [25], implying that conversion of the hydrogen bond network into vdW contacts may be responsible for reduced interaction with the virus.

Here we report the crystal structure of the complex between the engineered LFA-1 I domain and ICAM-1 D1. Distinct from the previous studies [17], [20], [26], we used a high-affinity I domain mutant with one substitution (F265S), while preserving the native sequence for the residues that are within or in direct contact with the α7-helix. Despite the relatively low resolution of the structure, we were able to establish that the α7-helix of the I domain in complex with ICAM-1 D1 was indeed displaced downward, comparable to the open conformations previously observed in α_M_ and α_2_ I domains [27], [28]. This recapitulates structural, allosteric linkage between the MIDAS and the position of α7-helix. Furthermore, the backbone structure of bacterially-expressed ICAM-1 D1, which contains many mutations and is devoid of molecular contacts with D2, was found to be closely superimposable to the previously solved D1 structures within D1D2 fragments expressed in mammalian systems.

## Results

### Structural Evidence for Allosteric Linkage between the MIDAS and the Position of the α7-helix in the LFA-1 I Domain

**Figure 1:**
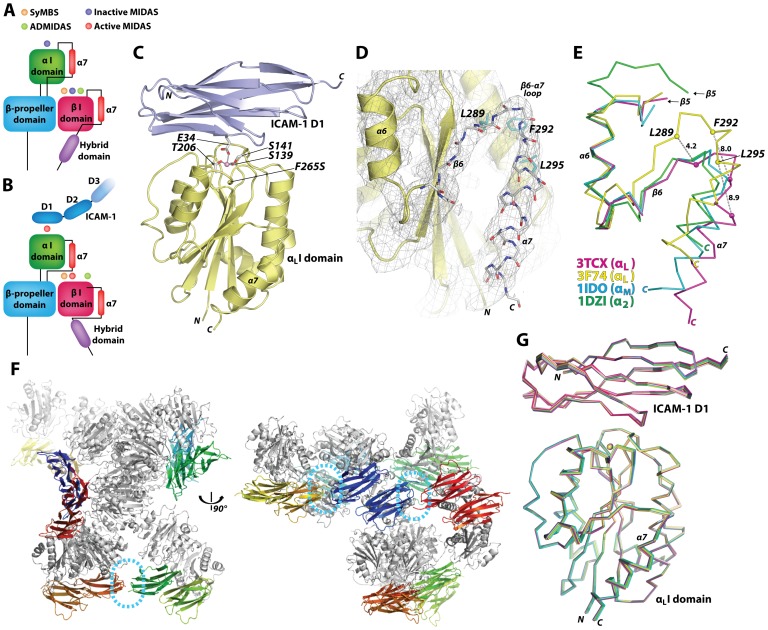
Complex structure of engineered α_L_ I domain and ICAM-1 D1. (A&B) Schematic drawings of the integrin headpiece, denoting intra- and inter-domain rearrangements during the engagement of LFA-1 with ICAM-1. A structural transition from low (A) to high affinity conformation (B) involves downward displacement of the α7-helix, shape change in metal-ion coordination sites, and swing-out movement of the hybrid domain. SyMBS = synergistic metal binding site; MIDAS = metal-ion dependent adhesion site; ADMIDAS = adjacent to MIDAS. (C) Ribbon diagram of the engineered α_L_ I domain (pale yellow), containing a substitution of F265S, in complex with the engineered domain 1 (D1) of ICAM-1 (light purple). The residues coordinating to the metal ion in the MIDAS, Ser-139, Ser-141, and Thr-206 in I domain and Glu-34 in ICAM-1 D1 are shown in stick models. The Mg^2+^ ion is shown as a pink sphere. (D) The electron density map, drawn together with cartoon or stick models, shows an open conformation of the β6-strand and the α7-helix. The three hydrophobic residues (Leu-289, Phe-292, and Leu-295; cyan) are shown in stick models. (E) In comparison to the previous open structures of the I domains of different α subunits, α_M_ (1IDO; blue) [27] and α_2_ (1DZI; green) [28], the α7-helix in our structure (3TCX; magenta) shows a comparable extent of downward displacement, away from the closed structure seen in the wild-type α_L_ I domain (3F74; yellow) [44]. (F) Ribbon diagrams of 14 complexes found in an asymmetric unit. I domains are drawn in grey, and 14 molecules of D1 are drawn in different colors for clarity. Dotted circles in cyan color indicate the interface between two C-terminal ends of D1. (G) Superimposed 14 complexes are shown as Ca-traces.

**Figure 2:**
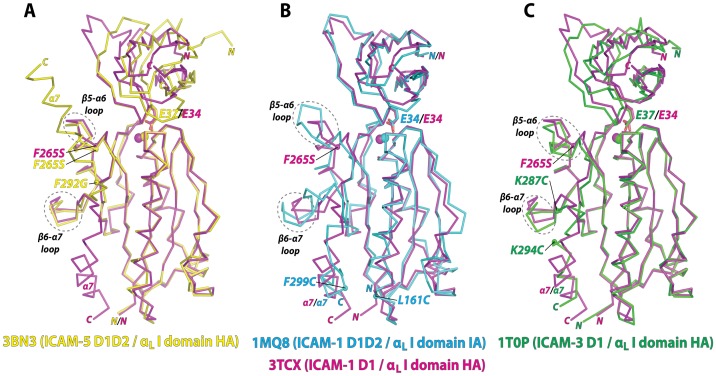
Comparison with the previous complex structures of the α_L_ I domain with ligands. (A–C) Superimposed to the current α_L_ I domain and ICAM-1 D1 structure (3TCX; magenta) are the previously solved complex structures of (A) high affinity (HA) α_L_ I domain containingF265S/F292G with ICAM-5 D1D2 (3BN3; yellow) [26], (B) intermediate affinity (IA) α_L_ I domain containing L161C/F299C with ICAM-1 D1D2 (1MQ8; blue) [17], and (C) high affinity (HA) α_L_ I domain containing K287C/K294C with ICAM-3 D1 (1T0P; green) [20]. The acidic residue of the ICAMs (Glu-34 in ICAM-1 and Glu-37 in ICAM-3 and ICAM-5) docking into the I domains and the Mg^2+^ ions are shown as stick and spheres, respectively. The β5-α6 and β6-α7 loops are circled with dotted lines.

Molecular contacts with the ligands by integrins, which contain the inserted or I domain in the α subunit, are contained solely within the I domain. In an inside-out signaling, a cascade of intra- and inter-domain conformational change occurs that propagates intracellular signals to ultimately the activation of the α I domain: a transition pathway follows the separation of α and β subunits at the plasma membrane, swing-out motion of the hybrid domain, activation of the I domain present in β subunit, and a final step of the activation of I domain in the α subunit (Fig. 1A&B). In the process of integrins engaging with their ligands, the downward ‘pull’ of the α7-helix located at the C-terminal end of the α I domain switches the MIDAS from low to high affinity conformation. To obtain a complex structure of the I domain with the ligands, α_L_ I domain, which maintains a modular function in isolation, has been engineered to high affinity by mutations, such as a pair of cysteines (K287C/K294C) to lock the α7-helix in an active conformation [17] or double mutations (F265S/F292G) that were identified by a directed evolution approach [22]. In an attempt to examine a physiologically attainable, high affinity conformation of the α7-helix with the least amount of perturbation, we chose the I domain with single substitution of Ser for Phe-265, a position located within β5-α6 and is not in direct contact with the residues in the α7-helix (Fig. 1C & Fig. 2). ICAM-1 D1 was previously engineered with seven mutations to achieve a native fold on its own with the affinity to the I domain comparable to that of the wild-type ICAM-1 D1D2 or D1-D5 [25]. The complex structure shows docking of Glu-34 in ICAM-1 D1 to a divalent metal ion (Mg^2+^) of the MIDAS (Fig. 1C), identical to the previous integrin-ligand complex structures [17], [20], [26], [28]. Even with the native sequence in the α7-helix and its preceding β6-α7 loop, the I domain was found in an open conformation with the α7-helix positioned downward, away from the closed state found in the wild-type (Fig. 1D&E). The backbone structure of the β6-strand and the α7-helix shown with electron density was found in the open state (Fig. 1D). The β6-strand and the α7-helix contain three hydrophobic residues, Leu-289, Phe-292, and Leu-295, which in concert determine the position of the α7-helix (open, intermediate, and closed) and the corresponding low or high affinity conformations of the MIDAS. Compared to the previous structures of the α_M_ and α_2_ I domains, the α_L_ I domain in our crystal structure exhibited a comparable extent of downward displacement of the α7-helix (Fig. 1E).

In contrast to one or two ICAM-1 D1D2 molecules or complexes per asymmetric unit (1MQ8, 1IAM, and 1IC1), notably a total of 14 complexes per asymmetric unit with a large unit cell dimension were found in our structure (Fig. 1F & Table 1). This may be partly ascribed to the interaction between two D1 fragments at the C-terminal face (indicated with dotted circles in Fig. 1F), which is naturally buried if D2 is present. However, the structural deviation (root-mean-square deviation (RMSD)) of all 14 complexes from each other was less than 0.41 Å. The largest deviations among the 14 complexes were found at the α7-helix of I domain (RMSD = 0.56 Å) and the loops connecting β-strands at the N-terminal face of ICAM-1 D1 (RMSD = 0.67 Å). The loops at the N-terminal face of ICAM-1 are also the regions that varied most among all previous crystal structures either in complex with α_L_ I domain or on its own (Fig. 1G).

**Table 1:** Data Collection and Refinement Statistics.

Space group	P2_1_2_1_2_1_
a (Å)	104.0
b (Å)	166.3
c (Å)	299.4
Molecules/asymmetric unit	14
Resolution (Å)	50-3.6 (3.66-3.6)
Unique reflections	58846 (2499)
Completeness (%)	96.2 (83.9)
R_sym_ (%)	12.4 (48.6)
<I/σ(I)>	14.6 (1.7)
Redundancy	5.5 (2.2)
R_work_/R_free_ (%)	21.8/23.4
Ramachandran Plot (favored/allowed/outlier %)	89.74/8.05/2.22
**Average B factor (Å^2^)**	
ICAM-1 D1	120.3
LFA-1 I domain	185.6
ICAM-1 D1+ LFA-1 I domain	165.7
**RMSD from ideal values**	
Bond lengths (Å)	0.013
Bond angles (°)	1.418

Number in parentheses are for the highest resolution shell.

### Comparison with the Previous Structures of High-affinity LFA-1 I Domain Variants in Complex with Physiologic Ligands

The LFA-1 I domain has previously been cocrystallized with ICAM-1 D1D2 [17], ICAM-3 D1 [20], and ICAM-5 D1D2 [26] (Fig. 2). All of the I domain structures were closely superimposable at the structurally invariant central β-sheet, with most deviations found in the β5-α6 loop, β6-α7 loop, and α7-helix. The affinity of the I domain to ICAM-5 was at least 10-fold weaker than to ICAM-1, and the I domain with two point mutations of F265S/F292G were necessary to form a stable complex for crystallization (Fig. 2A) [26]. However, the α7-helix was found flipped upward pivoting on Gly-292 in the β6-α7 loop and the vacated space was then occupied by the α7-helix belonging to a neighboring I domain within the crystal unit (Fig. 2A). This unnatural conformation of the α7-helix would be attributed to a greater flexibility in dihedral angles along the peptide backbone around Gly substituted for Phe-292. However, the backbone structure excluding the α7-helix (Asn-129 to Leu-289) of the F265S/F292G mutant was closely superimposable (0.6 Å RMSD) to the I domain containing only F265S. In complexes with ICAM-1 D1D2 and ICAM-3 D1, the I domains contained substitutions of two cysteines, which were introduced to lock the α7-helix in the intermediate (L161C/F299C) and the open (K287C/K294C) positions (Fig. 2B&C) [17], [20]. Compared to these disulfide bridge mutants, the β5-α6 loop (Gly-262 - Glu-272) of our structure was more closely superimposable to that in the high affinity mutant (RMSD = 1.8 Å) than that in the intermediate affinity mutant (RMSD = 3.1 Å) (Figures 2B and 2C). At the same time, the β6-α7 loop (Lys-276 - Val-286) of our structure was also more closely superimposable to the high affinity (RMSD = 1.5 Å to K287C/K294C) than to the intermediate affinity mutant (RMSD = 2.7 Å to L161C/F299C), implying that our structure adopted a high affinity conformation in the absence of mutations directly altering the position of the α7-helix.

### Structure of the Engineered ICAM-1 D1 Single Domain in Comparison with the Previous Wild-type Structures

**Figure 3:**
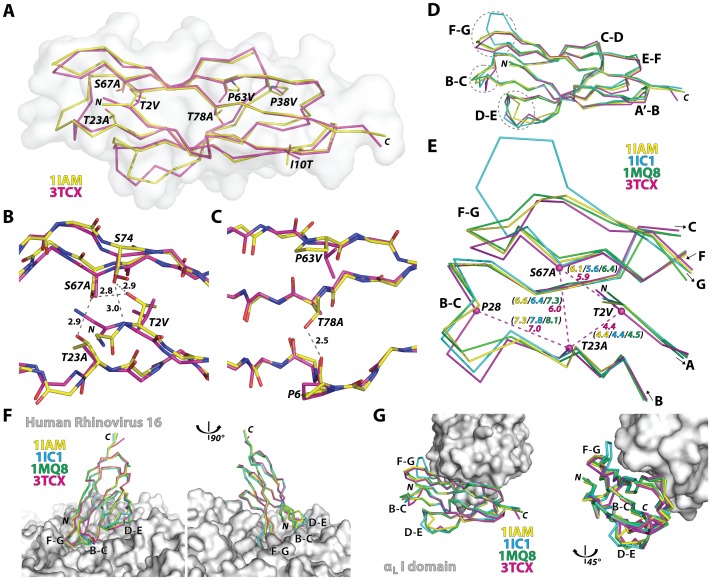
Structural deviation in engineered ICAM-1 D1 and its implication in ICAM-1 interaction with LFA-1 and HRV. (A) Cα-traces of engineered ICAM-1 D1 (magenta) and the wild-type D1 in D1D2 fragment (1IAM in yellow) are drawn with solvent-accessible surface plot (grey). (B) Hydrogen bond network formed by Thr-2, Thr-23, Ser-67, and Ser-74 at the N-terminal protein core is shown in grey dotted lines with distances in Å from the wild-type ICAM-1 structure (1IAM). Substitutions of T2V, S67A, and T23A in D1 are indicated. (C) A hydrogen bond between Pro-6 and Thr-78 is shown with a dotted line. Substitutions of P63V and T78A found in D1 are indicated. (D) D1 structures of the previous ICAM-1 D1D2 structures (1IAM in yellow [15], 1IC1 in blue [24], 1MQ8 in green [17]) were superimposed to engineered D1 (3TCX in magenta). The loops, B–C, D–E, and F–G, are circled with dotted lines. (E) The distances between the Cα atoms (dotted lines in magenta) of the triad Thr-2, Thr-23, and Ser-67, and the Cα distance between Pro-28 to Thr-23 in previous structures are compared with those in D1 mutant. (F) The superimposed ICAM-1 structures with the HRV were modeled based on the cryo-EM Cα coordinates of ICAM-1 D1D2 bound to HRV16 (1D3E [31] and 1AYM [45]). (G) The superimposed D1 structures are shown with the α_L_ I domain shown as solvent-accessible surface.

Unlike the retention of a modular function of the I domain on its own, functional expression of D1 by itself was achieved only after the introduction of seven mutations (T2V/A, I10T, T23A, P38V/A, P63V, S67A, T78A) into Gln1-Thr85 sequence, identified by the combination of directed evolution and rational design approaches [25]. Except for the mutations I10T and P38V, the remaining five mutations were located in solvent-excluded regions (Fig. 3A–C), converting polar residues into hydrophobic ones. Substitutions of T2V, T23A, and S67A would disrupt the hydrogen bond interactions near the N-terminal face of the domain and create hydrophobic, vdW contacts (Fig. 3B). Substitutions of P63V and T78A also create new vdW contacts in the protein core (Fig. 3C). For future structural studies with HRV, Gln-1 was mutated into Met to avoid an extra residue being appended to the N-terminal [29], which has been shown to grossly compromise ICAM-1 binding to HRV [30].

**Figure 4:**
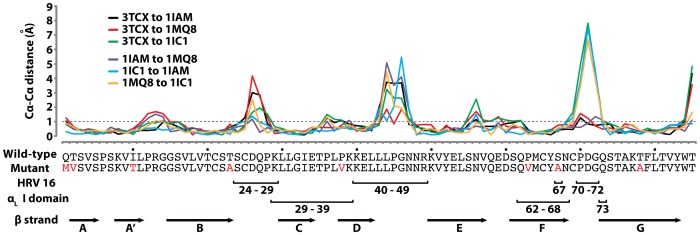
Differences in Cα positions for the superimposed structures of ICAM-1 D1. Pair-wise Cα-Cα distances between wild-type ICAM-1 (1IAM, 1MQ8, and 1IC1) and engineered D1 (3TCX) are plotted. Also, Cα- Cα distances among the wild-type ICAM-1 structures are plotted (1IAM to 1MQ8, 1IC1 to 1IAM, and 1MQ8 to 1IC1). Putative interacting residues of ICAM-1 D1 with HRV16 capsid as well as the residues in close contact with the α_L_ I domain are indicated with brackets and corresponding residue numbers. Arrow bars denote the residues that form the β-strands in ICAM-1 D1.

The backbone structure of the mutant D1 along the β-strands was closely superimposable to D1 structures in wild-type D1D2 fragments (0.5 Å RMSD to 1IAM & 0.7 Å RMSD to 1IC1) or in D1D2 in complex with the LFA-1 I domain (0.6 Å RMSD to 1MQ8) (Fig. 3D). The largest deviations of the mutant D1 from the wild-type structures as well as the largest among the wild-type structures were found at the F–G, B–C, and D–E loops that together create the contours of the N-terminal face (Fig. 3D & Fig. 4). These loops are in close contact with HRV as seen in the model structure generated from cryo-EM electron density (Fig. 3F) [31], [32], [33]. From our previous observation that the mutations into a triad forming a hydrogen bond network near the N-terminal face were responsible for the reduced binding to HRV [25], it can be speculated that our vdW forming mutations influenced the flexible nature of the N-terminal loops [24], which may be necessary for fitting into the viral capsid. We noted that Cα-Cα distances among Thr-2, Thr-23, and Ser-67 in the wild-type structures were slightly reduced in the D1 mutant with T2V, T23A, and S67A substitutions (Fig. 3E). Substitutions with smaller side chains (*i.e.*, T23A and S67A) in turn would have affected the interaction with the neighboring residues, thus placing the B–C loop closer toward the protein core (Fig. 3E). In contrast, the interface with the I domain is contained within the region where the backbone of engineered D1 was closely superimposable to that of the wild-type, unaffected by the deviation seen in the loops (Fig. 3G & Fig. 4).

## Discussion

Here we report the crystal structure of the complex of LFA-1 α I domain and ICAM-1 D1, which have been engineered for high affinity and native fold, respectively. A point mutation in the LFA-1 I domain (F265S), which resulted in an increase in affinity to ICAM-1 by ∼10,000 fold over the wild-type I domain [22], was sufficient to trigger allosteric shifting of the MIDAS into a high affinity metal ion coordination and a downward displacement of the α7-helix. Using a comprehensive, systematic approach to isolate mutations that would enhance protein fold, we have previously engineered a functional ICAM-1 D1 on its own, fully active in regard to its interaction with LFA-1 and less active with HRV [25]. Although as many as seven mutations were necessary to achieve functional D1 in isolation, our crystal structure revealed little perturbation in the conformation of the β-strands that form two faces of the β-sandwich structure. Despite extensive screening and optimization of crystallization conditions, the engineered complex in our study had repeatedly produced a large unit cell, and failed to diffract to high resolution that would greatly aid in more precise positioning of all the side-chains. However, all 14 complexes in the unit cell were highly superimposable with each other as well as to the previous crystal structures, validating the open conformation of the α7-helix of the I domain.

The structural basis of allosteric switching of the integrin I domains to the conformation that is competent for ligand binding has been studied extensively. Conformational allostery in the α7-helix was first evidenced in the α_M_ I domain structure with glutamic acid of the neighboring I domain coordinating to the metal ion in the MIDAS [27] and a concomittant downward displacement of the α7-helix. This was in contrast to later structures that revealed an upward position of the α7-helix and a lack of ligation to the MIDAS [34]. The first complex structure of the I domain with ligands was the α_2_ I domain bound to a collagen mimetic peptide, exhibiting a similar downward position for the α7-helix [28]. However, due to a low affinity of the wild-type LFA-1 I domain to ligands, previous complex structures with ICAMs required a pair of cysteines or the mutation (e.g., F292G) that would directly influence the position of the α7-helix. With the native sequence in the allosteric site around the α7-helix, the crystal structure of the complex in this study further underscores the intrinsic mobility of the α7-helix and how the I domain allostery is coupled to a global conformation change of integrins.

ICAM-1 consists of five Ig-like domains in its extracellular region, of which D1 is solely responsible for the molecular contacts with the LFA-1 I domain and HRV. Despite an extensive set of mutations in D1, which would convert mainly hydrogen bond interactions into vdW contacts, the D1 mutant was found to retain a native conformation of β-strands and β-sandwich fold with a largest deviation localized to the loops that form the N-terminal face of the Ig-like fold. Conformational variability of the loops in D1, analogous to the complementarity determining region (CDR) in antibodies [35], was also pronounced among the crystal structures of the wild-type ICAM-1 D1D2 with as much as 1.1, 2.9, and 4.8 Å RMSD between Cα distances in B–C, D–E, and F–G loops, respectively, whereas the rest of the domain differed by less than 0.6 Å RMSD. Although the stable D1 mutant retained its interaction with conformation-specific antibodies and the LFA-1 I domain, the full-length ICAM-1 D1-D5 containing the mutations found in D1 displayed lower binding to HRV [25]. In addition to the proposed role of charge complementarity at the interface between ICAM-1 and HRV [33], [36], the flexible nature of the loops [15], [24] and hydrogen bond network present in the N-terminal face of ICAM-1 may also be critical to recognition of over 90 different serotypes of rhinovirus by ICAM-1 [37]. The information gained from the crystal structure of the engineered ICAM-1 D1 and its comparison with the wild-type structures, *i.e.*, the importance of hydrogen bond network in the protein core that determines the conformation of the CDR-like loops for interaction with HRV, may provide a further insight into designing a functional D1 alone that retains full capacity binding to the virus. Functional D1, due to its low-cost production from bacteria, may be developed into a decoy antagonist to block LFA-1/ICAM-1 interactions and HRV binding to ICAM-1 expressing host cells.

## Materials and Methods

### Protein Production and Crystallization Condition

The LFA-1 I domain (Asn-129 - Tyr-307 with a mutation F265S) and ICAM-1 D1 (Gln-1 - Thr-85 with mutations Q1M, T2V, I10T, T23A, P38V, P63V, S67A, and T78A) were expressed in E. coli BL21 (DE3) (Novagen), refolded, and purified as previously described [25]. Equal molar of the I domain and D1 were mixed in the presence of 1 mM MgCl_2_ to form the complex, purified by size exclusion and ion exchange columns, and concentrated to 11 mg/ml in 50 mM HEPES, pH 7.2, 10% glycerol. Protein complex was then mixed at equal volume with the buffer for crystallization (0.1 M HEPES, pH 7.2, 10% glycerol, 0.4 M Na formate, 0.4 M NaCl, 22% PEG 6000, and 5% DMSO). Crystals were grown in a sitting drop at room temperature.

### Data Collection and Structure Refinement

The diffraction data were collected at Cornell High Energy Synchrotron Source (CHESS) Beamline F1 and processed with HKL2000 [38] for integration and scaling. The initial structural model was obtained by molecular replacement using PHASER [39] in CCP4 program suite [40]. There were a total of 14 complexes in an asymmetric unit and these complexes were found one by one [39] using the previous complex structures of ICAMs and α_L_ I domain (PDB codes 1MQ8 and 3BN3) as search models. Then the structures were refined with Refmac5 [41]. During refinement, 28 TLS (Translation/Libration/Screw) groups were assigned (14 groups for each ICAM (all atoms selected) and 14 groups for each α_L_ I domains (all atoms selected). Considering relatively low diffraction resolution (3.6 Å), tight non-crystallographic symmetry (NCS) restraints were applied to the 14 complexes. The refined structures were proofread and corrected with COOT [42] and refined again with Refmac5 until the crystallographic R and R_free_ factors converge to 21.8% and 23.4%, respectively. The structural refinement using PHENIX [43] produced essentially the same results with similar R and R_free_ values. The final structures were validated with PDB validation server (www.pdb.org). The coordinates of our complex structures have been deposited to the RCSB with the PDB code 3TCX. Details in data collection and structure refinement can be found in Table 1.

### Structure Alignments and Analysis

Previous structures of the α_M_ (1IDO) [27], α_2_ (1DZI) [28], and α_L_ (3F74) [44] I domains were superimposed to the α_L_ I domain of our structure (3TCX) based on residues in the α6-helix (α_M_: 278–288; α_2_: 294–304; α_L_: 268–278) (Fig. 1C). Previous complex structures of α_L_ I domain with ligands, which included ICAM-1 D1D2 (1MQ8) [17], ICAM-3 D1 (1T0P) [20], and ICAM-5 D1D2 (3BN3) [26], were superimposed to the α_L_ I domain of our structure (3TCX) using residues in the central β-sheet (129–140, 164–181, 231–237). Previous wild-type ICAM-1 D1 structures (1IAM [15], 1IC1 [24], and 1MQ8) were superimposed to the ICAM-1 D1 of our structure (3TCX) using the residues in the β-strands (2–5, 8–11, 15–23, 30–34, 38–42, 50–57, 61–68, and 73–83). A model structure of ICAM-1 D1 bound to HRV16 was constructed by aligning HRV16 coat protein (1AYM) [45] and ICAM-1 structures (residues 1–80) to the corresponding cryo-electron microscopy (cryo-EM) Cα coordinates of ICAM-1 D1D2 bound to HRV (1D3E) [31] (Fig. 3F). All the molecular graphic figures were made using PyMOL (DeLano, W.L.).
